# Rapid Particle Patterning in Surface Deposited Micro-Droplets of Low Ionic Content *via* Low-Voltage Electrochemistry and Electrokinetics

**DOI:** 10.1038/srep13095

**Published:** 2015-08-21

**Authors:** Noam Sidelman, Moshik Cohen, Anke Kolbe, Zeev Zalevsky, Andreas Herrman, Shachar Richter

**Affiliations:** 1 Department of Materials Science and Engineering Faculty of Engineering & University Center for Nano Science and Nanotechnology Tel Aviv University, Tel-Aviv, 69978, Israel; 2 Zernike Institute for Advanced Materials, Department of Polymer Chemistry, University of Groningen, Nijenborgh 4, 9747AG Groningen, Netherlands; 3 Faculty of Engineering and the Nanotechnology Center, Bar-Ilan University, Ramat-Gan, 5290002, Israel

## Abstract

Electrokinetic phenomena are a powerful tool used in various scientific and technological applications for the manipulation of aqueous solutions and the chemical entities within them. However, the use of DC-induced electrokinetics in miniaturized devices is highly limited. This is mainly due to unavoidable electrochemical reactions at the electrodes, which hinder successful manipulation. Here we present experimental evidence that on-chip DC manipulation of particles between closely positioned electrodes inside micro-droplets can be successfully achieved, and at low voltages. We show that such manipulation, which is considered practically impossible, can be used to rapidly concentrate and pattern particles in 2D shapes in inter-electrode locations. We show that this is made possible in low ion content dispersions, which enable low-voltage electrokinetics and an anomalous bubble-free water electrolysis. This phenomenon can serve as a powerful tool in both microflow devices and digital microfluidics for rapid pre-concentration and particle patterning.

Electrokinetic phenomena (EKP) are the induced movement of fluids, or of charged chemical entities immersed in them, due to an externally applied electric field^1^,^2^. Since their first discovery in the 19th century^3^, EKP have attracted considerable scientific attention, and have been extensively studied^1^. Nowadays, EKP are utilized in diverse fields of science and technology, to translate aqueous solutions and manipulate particles and molecules. Such uses range, e.g., from protein gel electrophoresis^4^, used in biochemical analysis, to industrial-scale electrophoretic deposition processes^5^ used for coating inorganic surfaces.

In recent years, the usage of EKP has expanded from macro-scaled applications (e.g. gel electrophoresis) to micro-scaled environments, i.e. to microfluidics^6^,^7^ and “lab-on-a-chip” devices^8^,^9^. EKP in macro-scaled applications is predominantly DC-based, and mostly relies on the classical DC-induced EKP of electroosmosis (EO) and electrophoresis (EP). However, in miniaturized devices the use of DC EKP is limited to one type of device only, in which liquid is translated along micro-channels^10^.

In theory, DC-induced EKP could serve as an excellent tool for liquid and particle actuation in micro-scale environments. This is since the magnitude of an electric field, which is the driving force in EKP, is inversely proportional to distance. Device miniaturization should have hence become an advantage. However, the use of DC EKP in modern miniaturized devices is limited due to an inherent effect that accompanies DC fields in aqueous media—electrochemical reactions at the electrodes. This drawback, which can be tolerated in macro-scale applications, can be detrimental for successful liquid and particle manipulation on the micro-scale.

The primary electrochemical reaction which hinders such successful manipulation in aqueous media is the electrolysis of water. Water electrolysis is comprised of oxidation at the anode 2*H*2*O*(*l*) → *O*2(*g*) 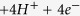 and reduction at the cathode 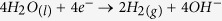
with an overall reaction of 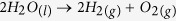. Because water electrolysis can begin at a voltage of as low as 1.23 V^11^ (though typically a voltage of 1.48 V is required^12^), electrolysis during DC EKP is practically unavoidable.

Water electrolysis impedes EKP in several ways. First, the evolution of oxygen and hydrogen gas bubbles mechanically hinders the migration of particles and molecules. Second, changes in pH, induced by the splitting of water molecules, adversely affect EKP, as it alters the zeta potential (ZP) of particles and substrate (i.e. EP and EOF velocities). Third, because water molecules are constantly decomposed, a large volume of liquid must be utilized.

DC EKP is used in microfluidic flow devices solely to translate liquids along long micro-channels^13^,^14^, because when utilized for that purpose, the adverse DC effects can be minimized. This is done by placing the electrodes in relatively large reservoirs, positioned far away from each other (several mm). The reservoirs not only allow large volume of solution to be used, but also locally confine pH changes to the reservoirs. More importantly, the reservoirs are fabricated in a way which ensures that the evolving gas is free to release^10^. This way, EKP in the channel linking the reservoirs is minimally affected.

Non-flow digital microfluidic^15^,^16^,^17^ devices, which handle discrete droplets and their constituents, also rely on liquid and particle actuation tools. However, DC EKP, which can be used to some extent in micro-flow devices, are considered completely unsuitable for liquid and particle manipulation in digital microfluidic devices^18^. This is mostly due to DC-associated difficulties, as encountered in flow devices described above. In non-flow devices, unfortunately, the adverse DC effects can’t be circumvented, due to the small distance scales dictated by the size of the micro-droplets.

In such devices, the surface-deposited liquid micro-droplets are manipulated using non-EKP effects^17^,^19^. The manipulation of chemical entities within the micro-droplets, mainly the concentration or trapping of particles and molecules, is predominantly performed using non-linear EKP, specifically AC-dielectrophoresis(DEP)^20^,^21^,^22^. DEP is an EKP that can be performed using AC fields, in which polarizable particles can be driven to locations corresponding to field minima or maxima in non-uniform electric fields. DEP is the tool of choice in digital microfluidics because electrochemistry can be avoided by the use of AC fields at sufficiently high frequencies.

Furthermore, DC EKP is presumably inherently limited, compared to AC-DEP, in the manipulation of particles in micro-droplets. Even if the abovementioned limitations of DC EKP could be overcome, DC EKP can still only be used to drive particles or molecules all the way to one of the electrodes (e.g., electrophoretic deposition), but not to any inter-electrode locations. This renders it useless for tasks such as particle concentration.

Here we show that contrary to the governing view, DC EKP can be successfully used to manipulate particles inside surface-deposited micro-droplets. We further demonstrate that DC EKP can induce particles not only to assemble in inter-electrode locations, but in fact to rapidly form patterns inside the micro-droplets. We show that the shape of the patterns, which do not correspond to field minima or maxima, can be manipulated in real-time. We further suggest that that the possible shapes of the particle patterns in each experimental setup can be predicted and hence be used for various purposes. We demonstrate that such particles patterning in open systems is made possible only in low conductivity dispersions, and results from a combination of low-voltage electrochemistry and low-voltage EKP. We further demonstrate that the occurrence of water electrolysis, which is viewed as a hindrance in DC-EKP, is essential for such particles manipulation owing to an anomalous bubble-free electrolysis. Such rapid particle enrichment/patterning can be highly useful in digital microfluidics since it can be performed using portable low-voltage DC sources rather than large AC sources, required for AC-DEP.

## Results

**Figure 1:**
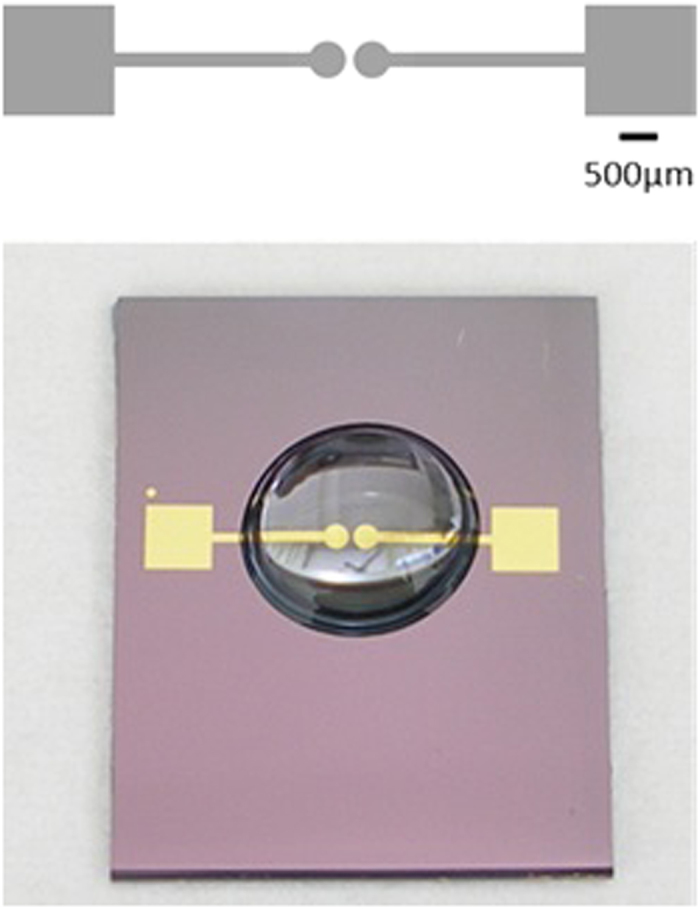
A scheme of the device (top) and an actual device with a 30 μl droplet deposited on it (bottom).

The device used in the following described experiments consists of a pair of identical 150 nm-thick gold electrodes, defined *via* photolithography on a silicon substrate with a 1000 nm-thick insulating oxide layer. The device’s area of interest is a junction, 100 μm wide at its narrowest region, defined by the electrode pair (see Fig. 1).

The experiments were conducted under a microscope equipped with a video camera, in order to record real-time particles behavior. The optical axis was perpendicular to the devices’ plane, so that the junction area was viewed from above, through the transparent dispersions.

### Particle Patterning in Micro-Droplets of Low Ionic Content

Particle patterning in surface-deposited, low ionic content micro-droplets is demonstrated below with titanium dioxide (TiO_2_) particles which could be continuously monitored by means of optical microscopy. 100 nm in diameter TiO_2_ particles were dispersed in ddH_2_O to yield a dispersion with a final concentration of 125 μg/ml. The dispersion’s conductivity was measured to be 4.0 μS/cm, and its pH slightly acidic, similar to that of ddH_2_O. The ZP of the dispersed TiO_2_ particles was measured (Malvern’s Zetasizer), and under the assumption of a large Debye length^2^ (κa << 1), was determined to be −19.2 ± 3.95 mV.

**Figure 2:**
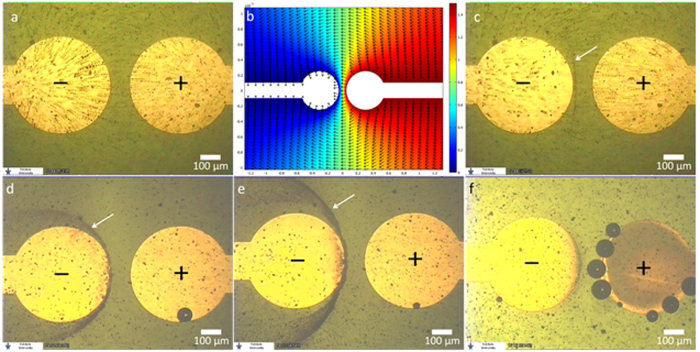
(**a**) Image comprised of 8 superimposed video frames showing electrokinetic particle trajectories between the positive pole (right) and negative pole (left), recorded at 1.5 V. (**b**) The simulated 2D potential map at an arbitrary voltage of 1.5 V in vacuum. The direction of electric field norm is indicated by the arrows. (**c**) Image comprised of 8 superimposed video frames showing the beginning of particle patterning (indicated by the arrow) in the inter-electrode region. (**d**,**e**) The stabilized patterns at applied voltages of 1.5 V and 2.0 V respectively. (**f**) Eectrochemical gold stripping and Cl_2_ gas bubbles observed in a dispersion droplet containing NaCl, at an applied voltage of 2.3 V. (See text for details).

A 30 μl aliquot of the dispersion was deposited on the device and DC voltage was applied. At a threshold voltage of 1.5 V (in the case of the TiO_2_ dispersion), a discernible continuous movement of the particles, from the positive pole to the negative pole, was recorded. Figure 2a is an image comprised of eight superimposed video frames, demonstrating particles trajectories. The first frame in the stack was acquired the moment 1.5 V were applied, and the time-gap between the first and last frames in the stack is 5.3 s. Figure 2b is the simulated 2D potential map and field direction at the substrate’s plane, in vacuum, at an arbitrary voltage of 1.5 V. It was obtained by solving Poisson’s equation for the device’s geometry using suitable boundary conditions. As can be seen, the recorded particle trajectories are consistent with the shape of the electric field.

A few seconds after particles movement began, they started to pattern inside the micro-droplet, a short distance away from the negative electrode (see Supplementary Movie 1). Figure 2c is an image comprised of ten superimposed video frames showing the beginning of particle trapping/patterning (indicated by a white arrow). The first frame in the stack was acquired ~14 s after voltage was applied, and the time-gap between the first and last frames is 6.7 s. Patterning was visible within ~12 s from the moment 1.5 V were applied. As can be seen, particle trajectories, which begin near the positive pole, end at the forming pattern.

As time progressed, the pattern changed position, stabilized and became more distinct, as more particles became trapped. Figure 2d shows such a stabilized TiO_2_ particles pattern, formed at an applied voltage of 1.5 V, 3 min and 56 s after voltage was applied. As can be seen, the stabilized pattern resembles a low-energy equipotential line (compare to Fig. 2b). Notably, the particles pattern could be manipulated in real time; when the voltage was increased to 2.0 V, the pattern began to change its shape almost instantly. Particles comprising the pattern began to move away from their original position, and the newly formed pattern stabilized within approx. a minute. This newly stabilized pattern, which resembles a higher-energy equipotential line, can be seen in Fig. 2e (see also Supplementary Movie 2).

### Particle Behavior in ion-rich Micro-Droplets

A TiO_2_ dispersion, similar to that described above, was spiked with NaCl, to reach a conc. of 100 mM. When a droplet of that dispersion was deposited and voltage was applied, neither particle movement nor patterning was recorded at 1.5 V or even 2.0 V. However, at 2.3 V both electrode stripping and Cl_2_ bubbles were observed, as shown in Fig. 2f.

The effect of ion content on the occurrence of low-voltage EKP and particle patterning was further evaluated using a non-chloride ionic salt. This is since chloride ions can be oxidized to Cl_2_ gas at 1.36 V. In addition, chloride ions promote galvanic gold stripping *via* the formation of a soluble tetrachloro-gold complex^23^. Hence, four identical TiO_2_ dispersions were spiked with increasing amounts of sodium nitrite, NaNO_2_, a 1:1 electrolyte, at final concentrations of 1.0 · 10^−5^ M, 1.0 · 10^−4^ M, 1.0 · 10^−3^ M and 1.0 · 10^−2^ M. The dispersions conductivities were measured to be 7.7 μS/cm, 14.9 μS/cm, 124 μS/cm and 1080 μS/cm respectively. As expected, the particles’ ZP decreased with the increase in salt content. In the dispersions spiked with 1.0 · 10^−5^ M and 1.0 · 10^−4^ M NaNO_2_, ZP was measured and determined to be, assuming κa << 1, −13.48 mV and −11.29 mV respectively. The ZP distribution in both cases was relatively large, and reached ±6.80 mV and ±7.59 mV respectively. This is attributed to the fact that the TiO_2_ particles comprised two crystalline polymorphs—rutile and anatase, whose point of zero charge differs by as much as 0.5 pH units^24^. It is likely that particle clustering further contributed to the relatively large distribution. In the dispersion spiked with 1.0 · 10^−3^ M and 1.0 · 10^−2^ M of NaNO_2_, the ZP could not be determined due to dispersions instability and rapid sedimentation, suggesting ZP is closer to zero.

The EK behavior and occurrence of particle patterning differed between the dispersions spiked with increasing amount of ionic salt (see Supplementary Figure 1). In the dispersions containing 1.0 · 10^−5^ M and 1.0 · 10^−4^ M NaNO_2_, at 1.5 V, particles moved in trajectories consistent with the shape and direction of the field. Rapid particle patterning took place as well, as in the non-spiked dispersions. In the dispersions containing 1.0 · 10^−3^ M and 1.0 · 10^−2^ M NaNO_2_, significant particle sedimentation was observed in the surface-deposited droplets. At 1.5V only the motion of particles located high above the substrate inside the micro-droplet was observed. The trajectories of these particles were mostly linear, and were inconsistent with the field shape. In addition, no particle patterns were observed at any voltage.

Patterning of particles in low-conductivity dispersions, similar to that of TiO_2_, was recorded with other particles as well, of different chemical composition and size (760 nm in diameter polystyrene particles, 300 nm silica particles, 9 μm alumina particles and 13 nm alumina particles). In all cases, particle patterns resembled equipotential lines and responded to voltage change. In each case, however, pattern location slightly differed (see Supplementary Figure 2).

It should also be noted that, in all cases, when voltage was decreased below the threshold voltage, or discontinued completely, the patterns quickly disintegrated.

## Discussion

The recorded particles behavior is an exceptional EKP at low DC voltages, over short distances inside a micro-droplet. As discussed below, low ionic content is essential for the EKP and for the patterning since it enables EKP to take place at unusually low voltage, as well as facilitates an anomalous bubble-free electrolysis. Both effects are crucial for particle patterning.

**Figure 3:**
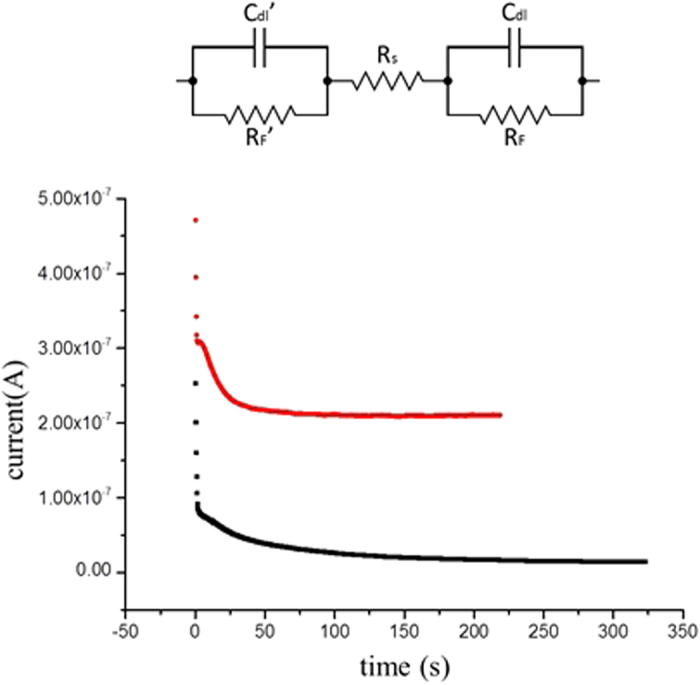
Top: The equivalent electrical circuit of a two-electrode cell. C_d_l and R_F_– the electric double layer capacitance and the faradaic resistance of the electrode-solution interface. Rs– the solution resistance. Bottom: The chronoamperograms of TiO_2_ particles in a droplet of ddH_2_O at 1.5 V (black) and 2.0 V (red).

As previously described, water electrolysis is considered an unavoidable hindrance in DC-EKP. Yet, some sort of an electrochemical reaction at the electrodes is essential for DC-EKP to take place. In order for long-lasting EKP to occur, there must be a sustained and significant voltage drop across the droplet. As the model of electrode-solution interface (Fig. 3 top) describes^11^, This can occur only when there is charge transfer across the interface, i.e. when a faradaic reaction occurs.

In the patterning experiments described above, electrokinetic particle motion was recorded, which indicates that a faradaic reaction took place. Yet, although the applied voltage exceeded the electrolysis threshold voltage, the videos and images acquired during experiments show that no bubbles evolved inside the droplet. As verified experimentally (see below), this is since an anomalous bubble-free electrolysis took place owing to the low ionic content of the dispersion.

A gas phase, i.e. bubbles, form inside a liquid phase when the liquid is saturated with the gas molecules^25^. Water in contact with air at atmospheric pressure requires a minute amount of dissolved H_2_ to become saturated, due to its low content in air. However, it is known^26^ that electrolyzed water can be super-saturated with molecular hydrogen, and dissolved H_2_ levels can be as high as 1.5 mg/L^27^. Because the solubility of O_2_ in water is significantly larger than that of H_2_^28^, and since O_2_ can also become super-saturated, during water electrolysis H_2_ bubbles evolve first, and in that sense, are the limiting factor. This is partly also due to the fact that its produced molar conc. is twice that of O_2_.

The anomalous bubble-free electrolysis in the low ionic media was made possible for several reasons. First, because in water rich with electrolytes, gas solubility is reduced^29^. Second, because the gaseous products of electrolysis can become super-saturated. Most importantly, large ionic content reduces the faradaic resistance and increases reaction rate. This is the reason modern electrolyzers, used to produce H_2_, are operated in ion-rich water^30^. The low ion content of the dispersion droplet ensures the electrolysis rate is minimized.

The amount of H_2_ (i.e. the limiting factor) produced during the particle patterning experiment can be estimated from the chronoamperograms (CA), collected during experimentation (Fig. 3 bottom).

Integration of current over time was performed on the CAs collected during the patterning experiments of TiO_2_ particles in pure ddH_2_O. At applied voltages of 1.5 V and 2.0 V for a duration of, e.g., 200 s, the integration reveals that the amount of charge which passed through the system is 6.56 · 10^−6^ C and 4.40 · 10^−5^ C respectively. Using Faraday’s law of electrolysis^25^, and assuming the only reaction which took place was electrolysis, at a 100% efficiency, this translates to 68.7 picogram and 460.6 picogram of H_2_ respectively.

Considering the super-saturation level that H_2_ can reach, it is clear that bubble-free electrolysis can take place in micro-droplets for a long duration at such current levels. Moreover, using the empirical Sechenov equation^31^, which describes the effect of ionic content on gas solubility in aqueous solutions, it can be found^29^ that hydroxyl ions only slightly reduce H_2_ solubility. For example, in extreme alkali conditions at pH 13, H_2_ solubility is reduced by about only 2%. Hence, although hydroxyl ions form during electrolysis at the same electrode as H_2_, gas solubility is marginally affected.

The low ionic content in the micro-droplets, which enables bubble-free electrolysis to take place, also has a direct effect on the electrokinetic aspect of particle patterning, and it facilitates low-voltage EKP and pattern formation. First, the low ionic content increases the absolute value of the particles’ and substrate’s ZP^32^,^33^. This means that under similar conditions, in ion-rich dispersions, stronger fields (higher voltages) are required to induce EKP, compared to low-ionic content dispersions. At such conditions, however, bubble formation is inevitable, and particle manipulation is rendered impossible. In addition, other gas-forming reactions, such as the oxidation of chlorine ions to Cl_2_ are avoided when ion levels are minimized.

Another direct effect of the low ion content on EKP relates to the magnitude of the electric field in the droplet. Shortly after voltage is applied and an electrochemical reaction begins, the only voltage drops between the poles of the DC source are across the electrode-solution interfaces (R_F_ and R_F_’, Fig. 3 top) and across the liquid droplet (R_S_). Ion content in the dispersion can affect the resistance of all three resistors.

In low ion-content dispersions (those prepared with ddH_2_O, and those spiked with 1.0 · 10^−4^ M and 1.0 · 10^−5^ M NaNO_2_), voltage drop across the solution is sufficient to induce EKP, so that particles follow electric field lines (and pattern). In the dispersions spiked with increased levels of ionic salt (those spiked with 1.0 · 10^−3^ M and 1.0 · 10^−2^ M NaNO_2_), results show that only particles above the substrate, which did not sediment, move in linear trajectories, and faster than particles in the low-ion content dispersions. The faster motion and the linear trajectories indicate that a larger field is exerted in the dispersions. This means that the excess ions in dispersion spiked with small amount of salt mostly reduce the faradaic resistance, so that the voltage drop across the solution is larger than in low ion-content dispersions. The linear trajectories arise because particles in those spiked dispersions acquire a sufficiently large velocity to cross the junction region without being significantly affected by the lateral forces there.

In very high ion-content dispersions (e.g. that spiked with 100 mM NaCl) the large excess of ions reduces the voltage drop across the droplet as well. Hence, higher voltages are required to induce particle motion. This, combined with the decrease in ZP, prevents particle movement with sufficiently low voltages.

**Figure 4:**
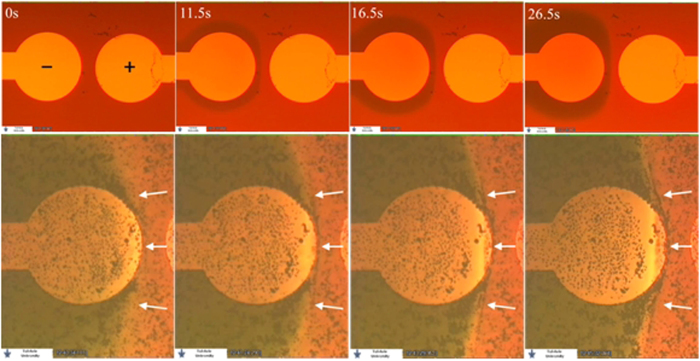
Top: the temporal-spatial evolution (left to right) of two pH zones in a Uind solution at 2.0 V due to a bubble free electrolysis (top). A basic, green region forms near the negative electrode and an acidic zone near the positive electrode can be seen). Bottom: temporal-spatial evolution (left to right) of stable pH zones, and particle patterning on the border of the pH zones. The border between the zones, where patterning occurs, is indicated by white arrows.

A significant factor, responsible for patterning, is that although bubble-free electrolysis takes place and bubbles do not evolve, pH changes at the electrodes still occur. The pH changes are the reason why particles slow down and pattern. The experimental evidence of the occurrence of pH changes is shown in Fig. 4 top. A 30 μl droplet of a universal pH indicator^34^ (Uind), which exhibits a broad color spectrum over a large pH range, was deposited on a device. The conductivity of the Uind solution was measured to be only 37.3 μS/cm, indicating that its ion content is low. Indeed, when 2.0 V were applied, bubble-free electrolysis was observed. The temporal-spatial evolution of the pH zones was monitored, and is demonstrated in Fig. 4 top (a comprehensive evolution of the pH zones is shown in supplementary Figure 3).

As can be seen, because there is no active mixing inside the droplet, two distinct pH zones rapidly form. A basic region around the negative electrode, and an acidic region around the positive electrode. It can be also seen that the shape of the border between the pH zones, during various stages of their temporal evolution, clearly resemble various equipotential lines.

The mechanism of particle patterning is hence suggested to be as follows: When voltage of at least 1.48 V is applied in low-ionic content dispersions, bubble-free electrolysis begins at the electrodes. The low ion level diminishes or eliminates completely other competing reactions. The voltage drop across the solution allows significant EKP to take place, and both EP and EOF begin. Initially EOF dominates over the EP, and the negatively charged particles move with the EOF in the shape and direction of the field, towards the negative electrode (see supp. videos). This is since the ZP of SiO_2_ substrate in contact with low-ionic content aqueous media^33^ can well exceed −100 mV (significantly above the particle’s ZP). A few seconds after voltage is applied, the concentration of hydroxyl ions increases near the negative electrode due to the bubble-free electrolysis. The ZP of particles crossing from the acidic zone into the basic zone changes sharply and becomes more negative, since the OH^−^ induces dissociation of protons from the particles surface groups. Because the particle ZP increases in absolute value, and since it has the same sign as that of the substrate, soon the EP velocity counter-balances the EOF thus particles settle and become quasi-stationary at equilibrium locations.

The experimental verification of the suggested patterning mechanism is shown in Fig. 4 bottom and in Supplementary Movie3. A solution of UInd was spiked with a dispersion of TiO_2_ particles similar to that described above. The ratio of ddH_2_O to UInd in the final dispersion was 1:50(v/v) and though particle sedimentation was significant it was still tolerable. A droplet of that dispersion was deposited on a device and voltage was applied. At 2.0 V, a change in the pH around the electrodes was observed. Due to the altered composition of the droplet, only at 2.5 V, the shape of the basic zone began to resemble the shape observed with the pure Uind. Almost simultaneously with the formation and stabilization of the pH zones, particles at the pH zones border started to become trapped along that border. Figure 4 bottom is comprised of 4 video frames showing the stabilization of the pH zones. Although many of the particles have sedimented quite early, it can clearly be seen in the image sequence (and supplementary movie3) that a particles pattern formed at the pH zones border as it stabilized, indicated by white arrows in the figure.

The possible shapes of the particles patterns, which resemble equipotential lines, can be predicted by finding the possible shapes of the pH zones. This can be done by considering the motion of H^+^ and OH^−^ which form at the opposite electrodes, and determining the location where they meet. Shortly after voltage application, the ionic velocities are time dependent. This is due to the pH changes which induces changes in the substrate’s ZP and hence EOF velocity (hence the experimentally observed changes in initial pattern shape). However, the stabilized shapes of the pH zones can be more easily determined by assuming a steady state has already been reached, i.e that the shapes of the pH zones are quasi-static and that two spatially stable pH zones have already formed. In such conditions, the ZP of the substrate no longer change over time, and is constant and uniform in each pH zones. In such a case, linear EKP can be assumed to govern in each zone separately. The H^+^ velocity in the acidic zone and the OH^−^ velocity in the basic zone can then be written as 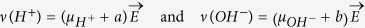, where *μ* is ionic mobility, E is the Electric field and *a*,*b* are constants representing the parameters in the EOF expression^1^. Since the substrate’s ZP differ between the acidic and basic zone, *a*≠*b*. Assuming that the concentration polarization (the increased excess of positive and negative ions in each zone) does not significantly perturb the electric field, possible shapes of the pH zones can be found by evaluating the motion of positive and negative test charges moving in an electric field at an arbitrary voltage. By evaluating the motion of each ion-pair on the various electric field lines, the shapes of the pH zone can be found. The charge ratio between a pair of positive and negative test charges then represents the ratio between the combined EP and EOF mobilities of the H^+^ and OH^−^ ions.

**Figure 5:**
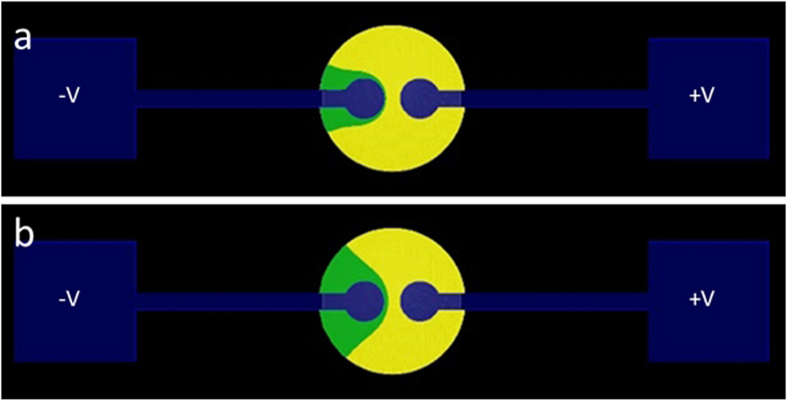
Numerical simulation of pH zones shape based on electrostatics at charge ratio of 11.7 (see text). The acidic zone is colored in yellow and the basic zone in green. Arbitrary voltages of (a) 1.5 V and (b) 2.0 V show the change in the zones shape as a function of field strength. See text.

Figure 5 shows the result of such a simulation performed for the case of the TiO_2_ dispersion previously described. It was obtained by a 3D numerical solution of Poisson’s equation out of which we extracted the 2D solution for the substrate plane (see SI data 1). A value was assigned to positive and negative test charges. The test charges were then “released” at the respective electrodes, and their movement was examined.

Figure 5 top shows where two such charges meet, and hence a possible shape of the pH zones, when the ratio between the charges was set to 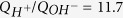. This ratio was found most suitable to represent the TiO_2_ experimental results previously described at an arbitrary simulated voltage of 1.5 V. The line on which the charges met is the border line between the two colored regions. The basic region around the negative electrode is colored in green while the acidic zone around the positive electrode is colored in yellow.

The charge ratio was set in favor of the charge representing H^+^ due to EP and EOF considerations; the ionic mobility of H^+^ (μ_H_^+^) is the highest of all ions. In water at 25 °C it is more than 1.7 times larger than that of OH^−^. This means that H^+^ electrophoretically migrates significantly faster than the hydroxyl ion. Moreover, the substrate’s ZP in the basic (green) region is highly negative, meaning that the EOF at that region is directed leftwards toward the negative electrode. The total velocity of OH^−^ is hence further decreased. The velocity of H^+^ is also slightly reduced, since in the acidic region, the substrate’s ZP is close to zero, and EOF towards the basic zone is decreased. Still, in such conditions, it is reasonable to assume that ν(H^+^) >> ν(OH^−^).

As can be seen in Figure 5a, at the chosen charge ratio, the border of the pH zones highly resembles a low energy equipotential line. In fact, it highly resembles the experimentally observed TiO_2_ pattern at 1.5 V shown above (compare to Figure 2d).

Figure 5b show the shape of the pH zones at the same charge ratio, at higher applied voltages (an arbitrary voltage of 2.0 V). The resemblance of the pH zones border to experimentally observed TiO_2_ patterns at the higher voltage can be clearly seen. As was observed experimentally, the simulation shows that when voltage is increased, the shape of the pH zones, and that of the particles pattern, change mostly at its flanks, and barely at the junction region (compare to Figure 2e).

The charge ratio which best represent the cumulative EP and EOF velocities of H^+^ and OH^−^ ions in the low conductivity TiO_2_ dispersion in contact with SiO_2_ was found to be 11.7 (for the arbitrary voltage and conditions used in the simulation). Ultimately, for any combination of a dispersion-substrate pair, empirical values can be found that best simulate the predicted change in the shapes of the patterns.

It should be noted that the patterning mechanism demonstrated above somewhat resembles the governing mechanism in equilibrium-gradient separation methods^35^,^36^, often employed in analytical science. In those methods, analytes travel down a linear separation path and stop when the net force acting on them vanishes (e.g. iso-electric focusing^37^). However, in the case of the currently described particles patterning, the particles are concentrated in a non-confined micro-droplet rather than in liquid inside a capillary or micro-channel. Moreover, it occurs due to a combination of low-voltage EKP and bubble-free electrolysis. An additional remarkable difference is the number of dimensions in which the manipulated entities are focused. Gradient focusing methods are employed only in linear separation paths. This means that the manipulated analytes are focused or trapped in a straight, one-dimensional band along the separation path. Here, focusing effectively occurs in 2D shape and the chemical entities are patterned in distinct spatial 2D “traps”, and not just focused in 1D.

In summary, we have shown that due to a unique combination of electrochemistry and EKP taking place in dispersions of low ionic content, particles within them can be manipulated and rapidly patterned/concentrated. The hindrances associated with DC EKP are circumvented due to the low ionic content; Bubbles do not evolve and do not hinder particle motion. The pH changes give rise to distinct pH zones and cause particles to settle in distinct location. In addition, the low current level and the fact that H^+^ and OH^−^ recombine on the border of the pH zones to form H_2_O mean that the droplet volume barely changes during experimentation. This phenomenon, which contradicts the governing view in applied EKP can serve as a powerful tool in various microfluidic applications which require rapid concentration. Since particle pattern shape is determined primarily by electrode geometry, the pattern shapes can be pre-planned and adjusted in real-time.

## Additional Information

**How to cite this article**: Sidelman, N. *et al.* Rapid Particle Patterning in Surface Deposited Micro-Droplets of Low Ionic Content *via* Low-Voltage Electrochemistry and Electrokinetics. *Sci. Rep.* **5**, 13095; doi: 10.1038/srep13095 (2015).

## Supplementary Material

Supplementary Information

Supplementary Movie 1

Supplementary Movie 2

Supplementary Movie 3
