# Supplementary material for: Rapid Particle Patterning in Surface Deposited Micro-Droplets of Low Ionic Content via Low-Voltage Electrochemistry and Electrokinetics
Source: Sci Rep. 2015 Aug 21;5:13095. doi: 10.1038/srep13095 (PMC4543935; doi:10.1038/srep13095)
Supplement: Supplementary Information [file srep13095-s1.pdf]

Low Ionic Content *via* Low-Voltage Electrochemistry and Electrokinetics

Noam Sidelman[1], Anke Kolbe[2], Moshik Cohen[3], Zeev Zalevsky[3] Andreas Herrman[2*] and Shachar Richter[1*]

[1]Faculty of Exact Sciences &University Center for Nano Science and Nanotechnology, Tel Aviv University, Tel-Aviv, 69978, Israel

Faculty of Engineering , Bar-Ilan University, Ramat-Gan, 5290002, Israel

Email: srichter@post.tau.ac.il

[2] Zernike Institute for Advanced Materials , Department of Polymer University of Groningen, Groningen, Netherlands.

Email: a.herrmann@rug.nl

Supporting Figure 1

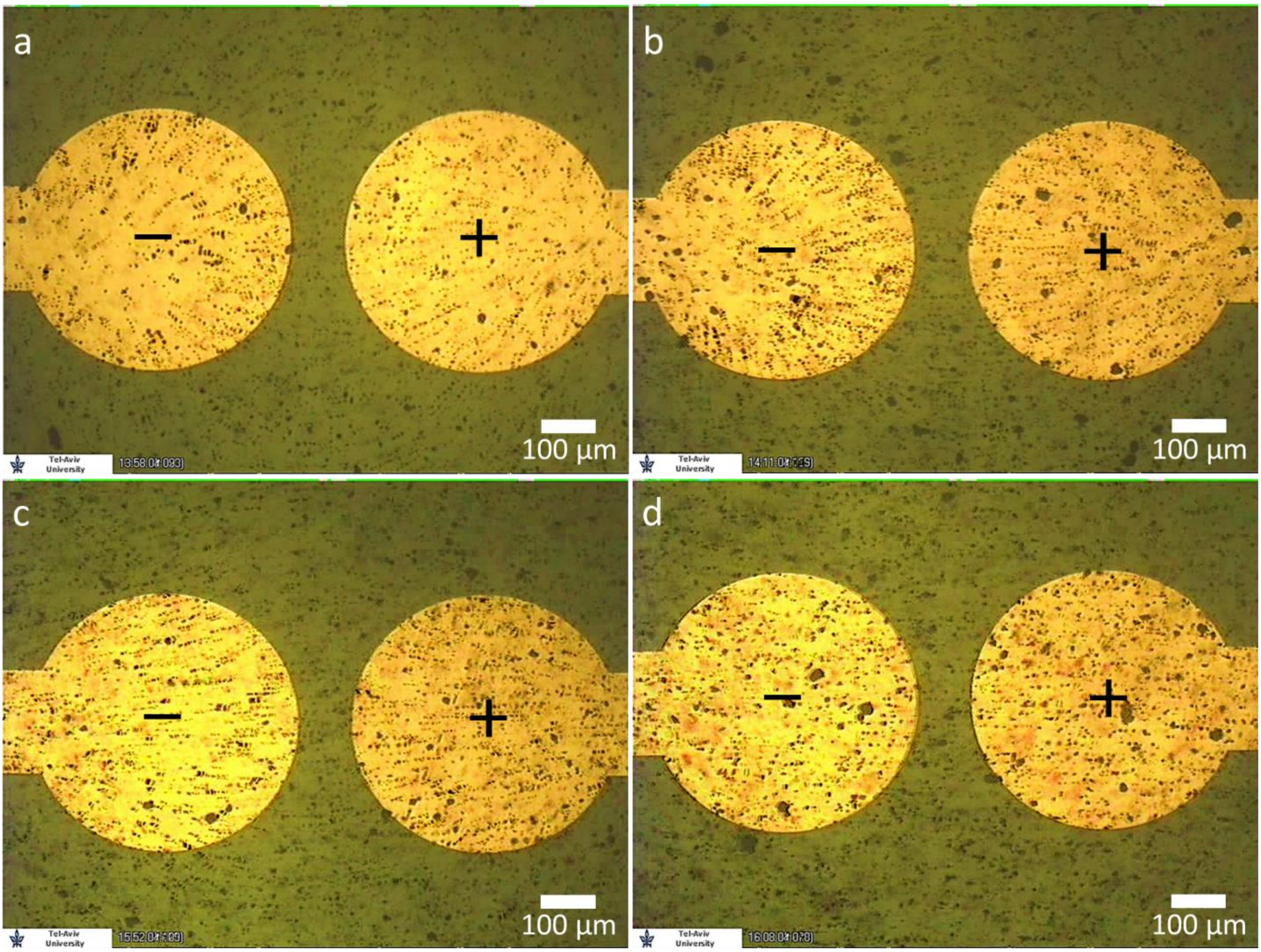

Particle trajectories at an applied voltage of 1.5V in the four dispersions spiked with $NaNO_2$ at conc. of. (a) $1.0\cdot10^{-5}$M **(b)** $1.0\cdot10^{-4}$M **(c)** $1.0\cdot10^{-3}$M **(d)** $1.0\cdot10^{-2}$M. Each images is comprised of five superimposed video frames, 1sec apart, beginning the moment 1.5V was applied.

Supporting Figure 2

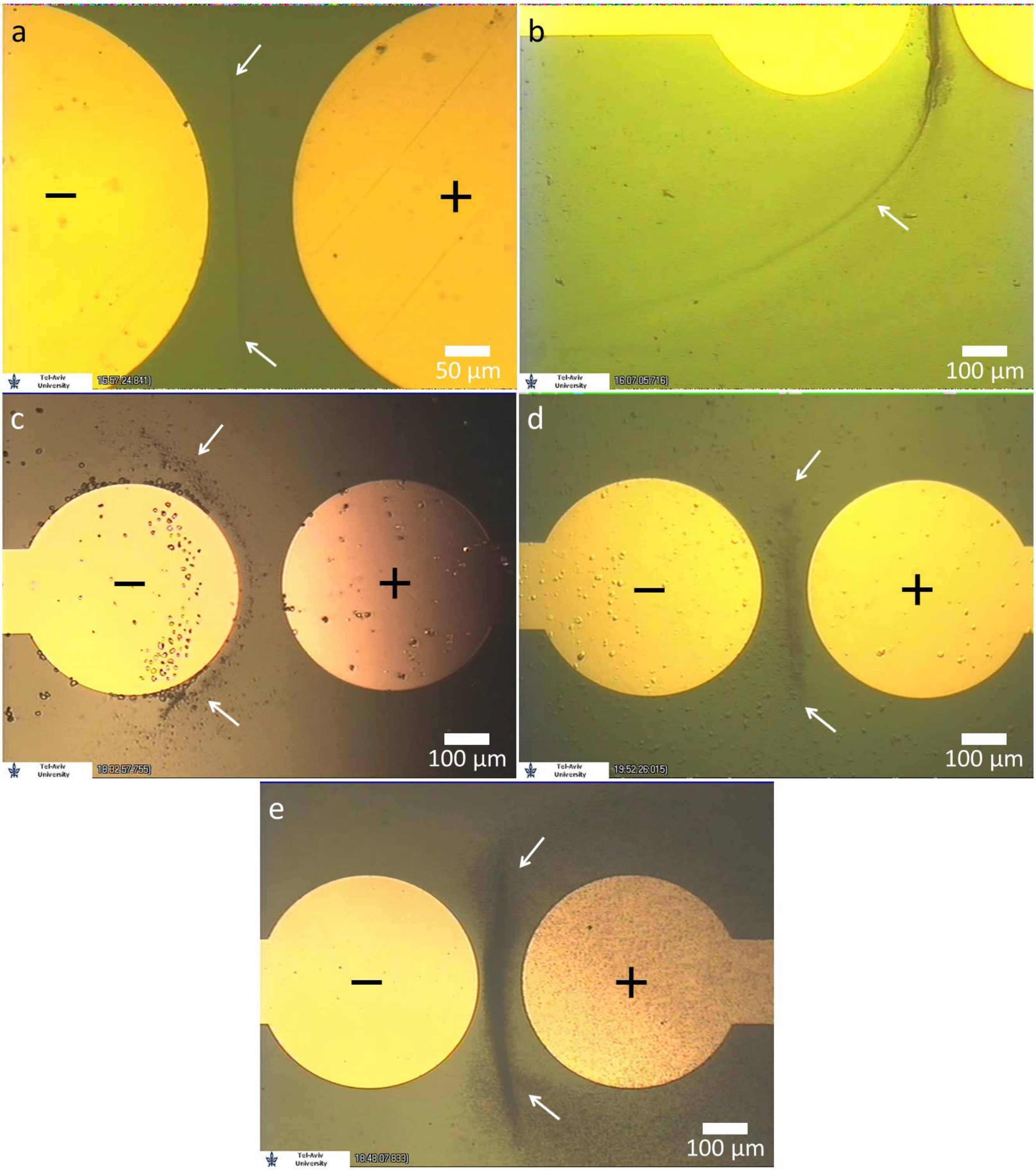

(a) Patterning of 300nm in diameter Silica particles. Unlike the TiO_2 particles, the silica particles began to pattern only at 2.0V**. (b)** The stable Silica particles pattern at 2.0V extended to significant distances away from the electrodes. **(c)** A pattern of 9µm in diameter Alumina particles at an applied voltage of 2.5V. **(d)** A pattern of 13nm in diameter Alumina particles at 2.0V. **(e)** Patterning of 760nm in diameter polysterene particles at 2.0V.

Supporting Figure 3

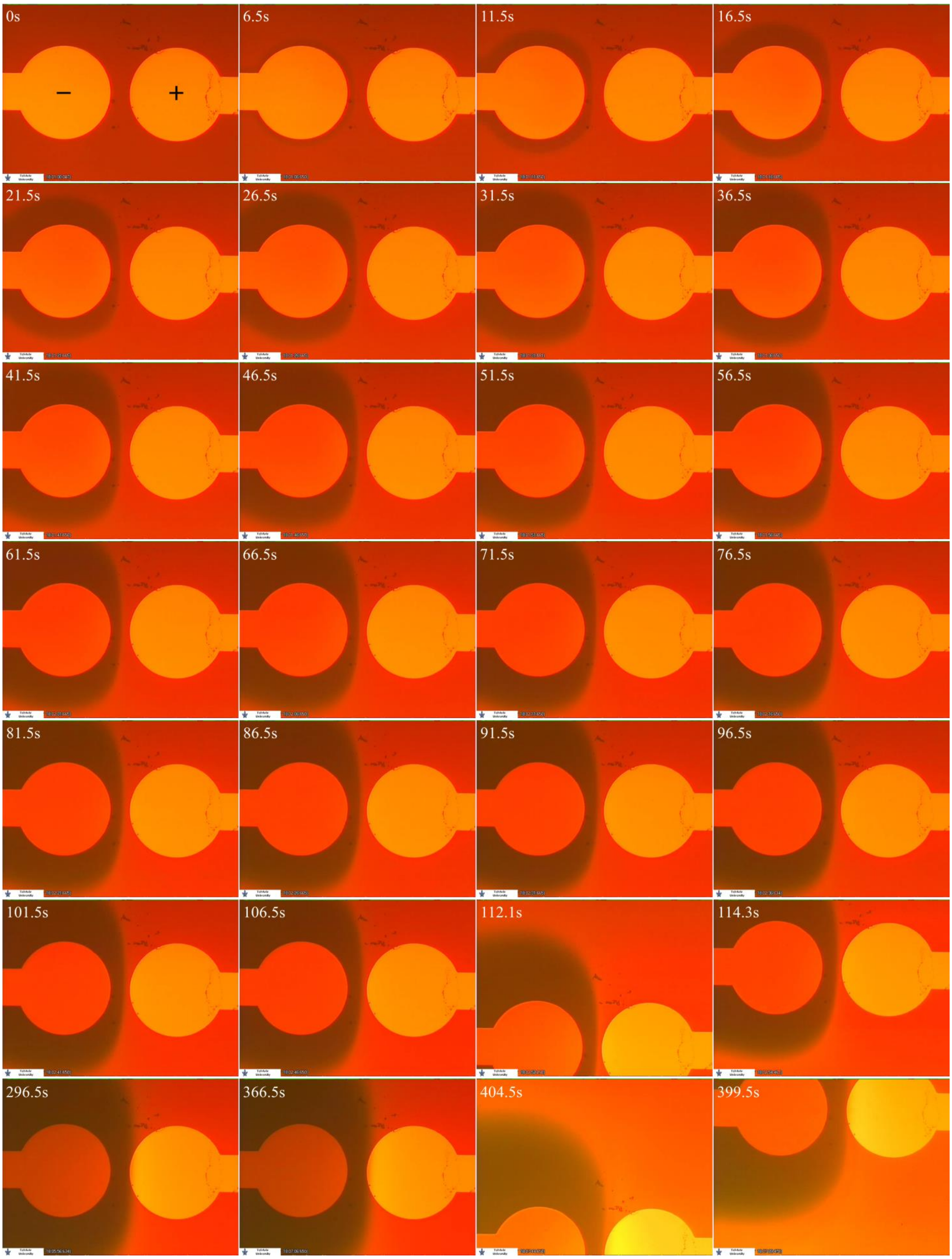

Temporal-spatial evolution of two pH zones, inside a 30µl droplet of a Universal pH Indicator, at an applied voltage of 2.0V. The image sequence clearly shows that the initial shapes of the basic green zone's border resemble equipotential lines. It can be seen that in this case, due to the composition of the droplet, the basic pH zone eventually drifts.

Supporting Data 1

Theoretical Analysis of Particles in Electrostatically Biased Water Droplet

The field solutions are calculated using full - wave finite-element field-analysis software, Maxell 3D v. 14, Ansys Electromagnetics [1–3]. The solution space is divided into a tetrahedral mesh and the potential is calculated at each vertex and the mid-point of every side from Poisson's equation. The potential is approximated by a low-order polynomial so that the electric field across each tetrahedron can be calculated, with the accuracy of the solution controlled by the number of elements in the mesh. Error analysis is performed; minimizing the energy functional in each tetrahedron. Subsequently, the mesh is refined at the regions of highest error with the number of mesh elements is increased until the required solution accuracy is reached. The model was fully analyzed in three dimensions without using symmetry, as shown in Figure 1a. Selectively dense meshing was assigned at the metal - water interface, with maximum mesh cell size of 1µm and total mesh cells of 150,000 see Figure 1b. The convergence criterion was set to less than 1% variation of the energy functional between three successive iterations of mesh refinement, assuring very high accuracy of the field solution. The structure is biased using DC voltage boundary ($\pm V_{DC}$) applied at the metallic ports, which modeled using DC conductivity of 41000000 Siemens/m.

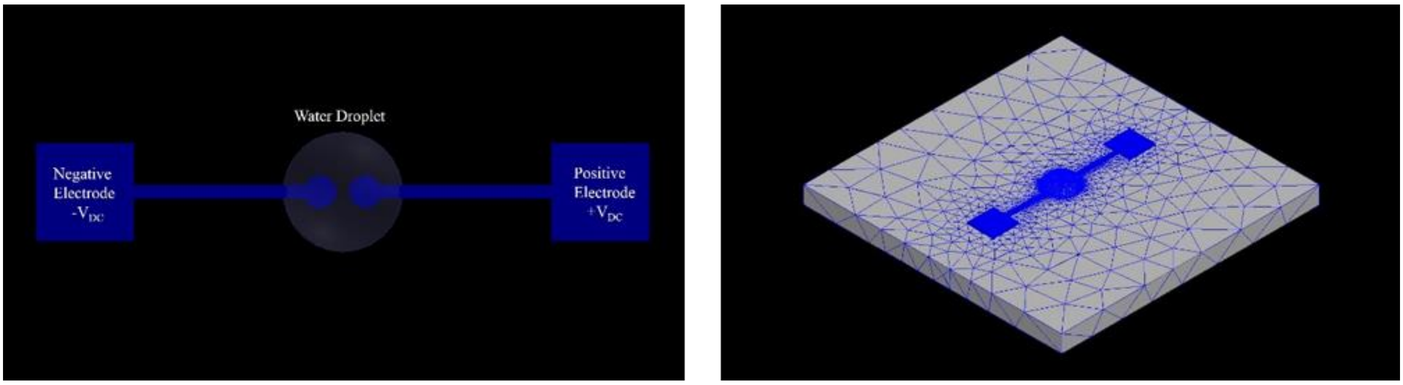

Figure 1|3D electromagnetic model a, Top view on the model **b,** Numerically obtained finite element mesh which used for the field solution. The meshing configuration includes 150,000 tetrahedral elements

Relative permittivity of 81 and bulk conductivity of 0.0002 Siemens/m were used to model the distilled water droplet, and the relative permittivity of SiO_2 at zero frequency is 4. From the 3D calculation which better represents the assymetrical nature of the problem we extracted the calculated electrostatic potential distribution on the plane of the substrate. This is shown on Figure 2a, for V_{DC} = 2.0V. The formation of pH zones at the steady state was modeled as the confluence curve of a positively and negatively charged particle moving from the positive and negative electrode, respectively. We considered a positive particle located at the + VDC electrode, and a negative particle located at the - VDC electrode.

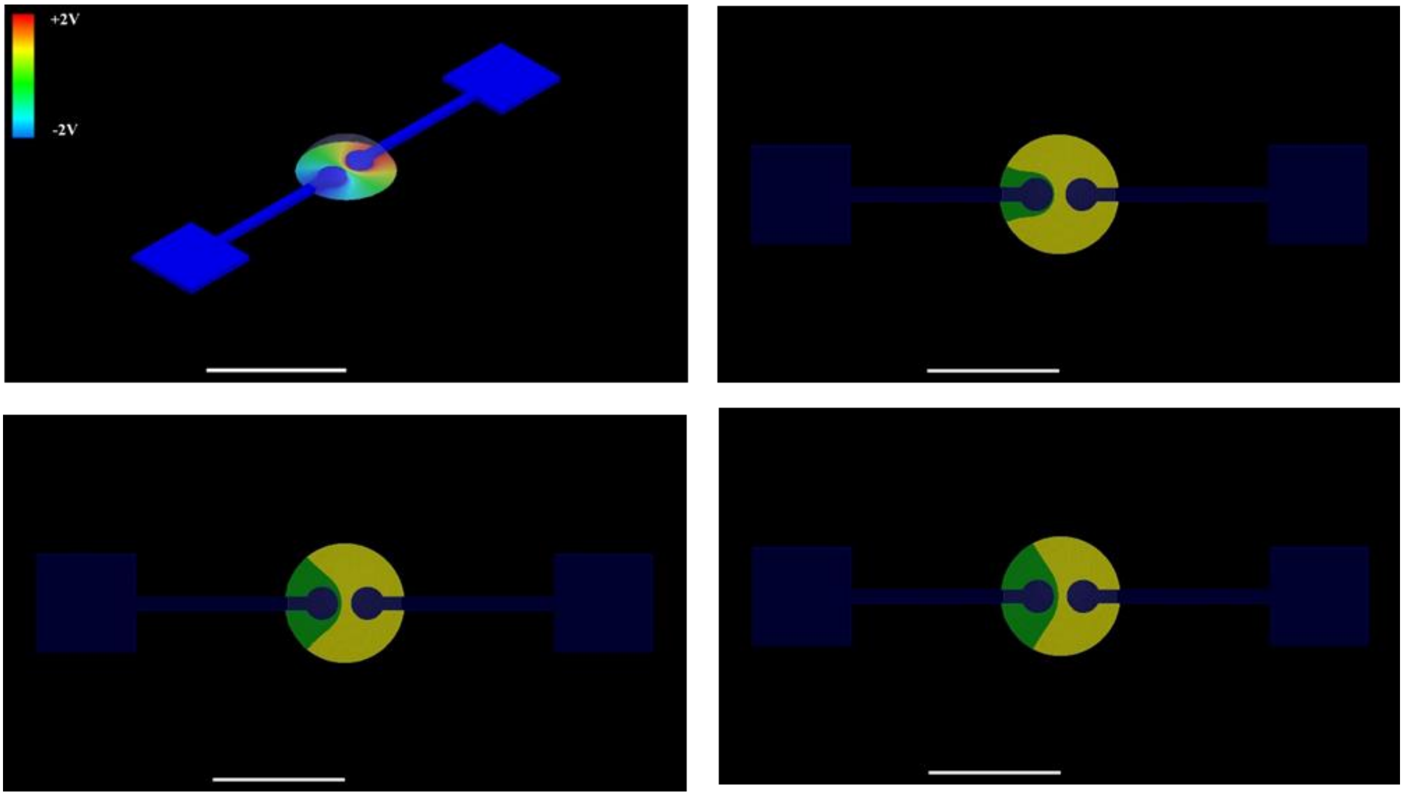

Figure 2| 3D Numerical simulation of the structure, with pH zones formation. **a,** 3D view of the electrostatic potential on the surface **b,** Numerically calculated pH zones formations for $\pm V_{DC} = \pm 1.5V$, surface **c,** Numerically calculated PH zones formations for $\pm V_{DC} = \pm 2.0V$, **d,** Numerically calculated pH zones formations for $\pm V_{DC} = \pm 2.5V$

References

[1] J. Voldman, R. A. Braff, M. Toner, M. L. Gray, and M. A. Schmidt, Biophys. J. **80**, 531 (2001).

[2] M. Cohen, Z. Zalevsky, and R. Shavit, Nanoscale (2013).

[3] B. P. Lynch, A. M. Hilton, and G. J. Simpson, Biophys. J. **91**, 2678 (2006).
